# Shared Genetic Architecture Between COVID-19 Severity and Alzheimer’s Disease Across European and African Ancestries

**DOI:** 10.21203/rs.3.rs-5619229/v1

**Published:** 2024-12-24

**Authors:** Jingchun Chen, Davis Cammann, Tingwei Liu, Yimei Liu, Melika Cummings, Xiangning Chen, Edwin Oh, Jerome Rotter

**Affiliations:** University of Nevada Las Vegas; University of Nevada Las Vegas; University of Nevada Las Vegas; The university of Texas Health Science Center at Houston; UNLV School of Medicine; The Lundquist Institute for Biomedical Innovation at Harbor-UCLA Medical Center

**Keywords:** Alzheimer’s disease, COVID-19, genome-wide association studies (GWASs), polygenic risk scores (PRSs), genetic correlations, APOE

## Abstract

The global outbreak of COVID-19, caused by the SARS-CoV-2 virus, has been linked to long-term neurological complications, including an increased risk of Alzheimer’s disease (AD) among older adults. However, the precise genetic impact of COVID-19 on long-term AD development remains unclear. This study leveraged genome-wide association study (GWAS) data and genotype data to explore the genetic association between AD and various COVID-19 phenotypes across European ancestry (EA) and African ancestry (AA) cohorts, and the possibility of a causal effect of COVID-19 on AD. We first calculated polygenic risk scores (PRSs) of three COVID-19 phenotypes in AD cases and controls from both EA and AA populations, then determined the genetic associations between COVID-19 PRSs and AD by logistic regression analyses with or without adjusting for age, sex, and *APOE* genotypes. Significant positive associations were found between AD diagnosis and COVID-19 PRSs in both populations, with the strongest associations identified in the AA population. However, Mendelian randomization (MR) analyses revealed no evidence of a causal effect of COVID-19 phenotypes on AD liability. We explored this finding further through the analysis of shared genomic regions between the COVID-19 phenotypes and AD and found a region of overlap on chromosome 17 that was highly pleiotropic for traits implicating immune function, psychiatric disorders, and lung function phenotypes. These findings suggest that while COVID-19 and AD share overlapping polygenic contributions involving peripheral genes across multiple traits, they lack a direct connection involving core genes that drive the development of their respective pathologies.

## Introduction

Alzheimer’s disease (AD), the most prevalent type of dementia, is a neurodegenerative condition defined by a combination of pathological and clinical features, including a gradual loss in cognitive function and the accumulation of toxic beta-amyloid and tau proteins in the brain (1, 2). The number of individuals with dementia is projected to reach 150 million globally by the year 2050 due to the growing elderly population worldwide (3). A limited understanding of AD etiology impedes the development of optimally effective treatments (4). The potential role of infectious agents in AD etiology remains an area of active investigation, with emerging evidence suggesting neurotropic pathogens may influence disease onset and progression (5). In this context, SARS-CoV-2 infection has emerged as a significant neurological concern, demonstrating both acute and chronic impacts on central nervous system function. Clinical studies indicate that approximately 33% of COVID-19 patients manifest neurological symptoms, ranging from mild manifestations (anosmia, dysgeusia) to severe complications (encephalopathy, stroke)(6). Along with the long-term effects on the respiratory system, growing evidence suggests that COVID-19 infection affects the central nervous system (CNS), which may result in long-term neurological sequelae, such as cognitive decline and dementia (7). While public health legislation and vaccines have slowed the COVID-19 outbreak, the long-term consequences of COVID-19 infection are becoming more widely acknowledged and worrisome. Several mechanisms have been proposed to explain the potential COVID-19 neurological injury that could lead to the development of neurodegenerative diseases, including AD.

SARS-CoV-2 infection triggers complex neuroinflammatory cascades characterized by systemic immune dysregulation, potentially mediating neuronal and microglial dysfunction through both direct cytopathic effects and indirect inflammatory mechanisms (8, 9). Epidemiological evidence demonstrates a bidirectional relationship between COVID-19 and AD pathogenesis: longitudinal analyses reveal significantly elevated risk of incident AD diagnosis among COVID-19 survivors, while pre-existing AD represents a prominent risk factor for severe COVID-19 outcomes and adverse clinical trajectories (10, 11). Despite these established clinical associations, investigation of chronic neurological sequelae has been less comprehensive than characterization of acute cardiopulmonary manifestations. The genetic architecture underlying susceptibility to both COVID-19 and neurodegenerative processes remains less understood, particularly regarding shared genetic determinants that may influence long-term neurological outcomes following SARS-CoV-2 infection. Systematic examination of genetic variants associated with both COVID-19 severity phenotypes and AD risk holds potential for elucidating fundamental biological pathways, thereby informing development of targeted therapeutic interventions and enabling identification of individuals at elevated risk for adverse neurological sequelae.

Polygenic risk scores (PRSs), which summarize the cumulative effects of genetic variants—typically single nucleotide polymorphisms (SNPs) identified in genome-wide association studies (GWAS) (12) are a powerful tool for predicting disease risk when the GWAS trait aligns with the target condition. Beyond direct prediction, PRSs are increasingly employed to explore genetic associations between distinct complex traits, even when the GWAS trait and the target condition differ. This approach has been successfully demonstrated in our previous work (13, 14) and studies by others (15, 16).

As PRS becomes more widely used, especially regarding their potential clinical utility, it is essential to ensure that every ethnic group can benefit from genetic studies. However, current genetic studies predominantly focus on populations of European ancestry (EA), which limits their applicability to those from non-European ancestries. In the present study, we aimed to evaluate the genetic relationship between AD and COVID-19 in both the EA and AA populations. For this purpose, we integrated AD genotype data and COVID-19 GWAS data with three COVID-19 phenotypes from the COVID-19 Host Genetics Initiative release 7 (HGI7), including Critical illness (A2), Hospitalization COVID-19 (B2), and COVID-19 infection (C2). Finally, we explored the causal effect of COVID-19 on AD using two-sample Mendelian randomization (MR) analyses.

## Materials And Methods

### 3.1 Study design

**Figure 1:**
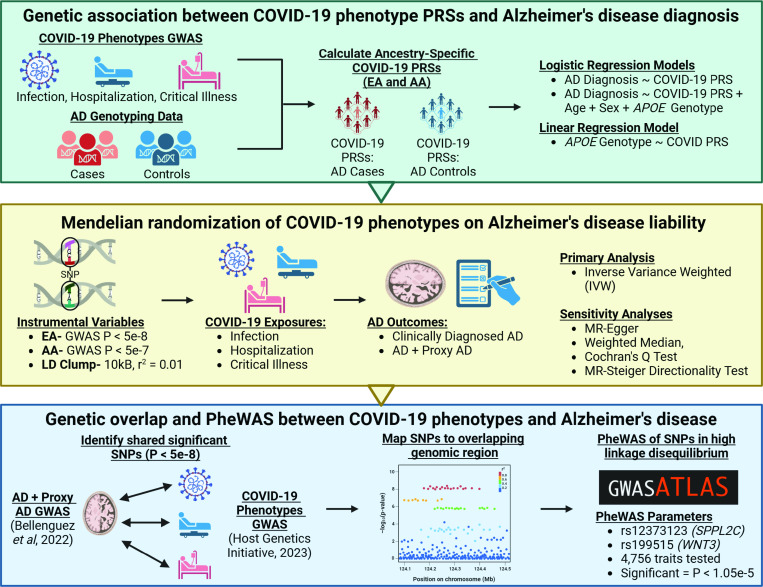
Study design flowchart We used three different phenotypes of COVID-19 from EA and AA populations to generate ancestry-specific PRSs of each phenotype in AD cohorts. We used logistic and linear regression models to explore the association between COVID-19 phenotype PRSs, AD diagnosis, and *APOE* genotype. Following this, we used each COVID-19 phenotype as an exposure in a two-sample Mendelian randomization analysis with AD liability as an outcome. Significant variants from GWASs of COVID-19 and AD were mapped and annotated to shared genes. We then conducted a PheWAS on SNPs found in the overlapping genomic regions between the COVID-19 phenotypes and AD. Created in https://BioRender.com

The study design is outlined in Fig. 1. Briefly, PRSice-2 (17) was used to calculate the PRSs for COVID-19 phenotypes in individuals from our AD datasets (target data), using GWAS summary statistics for three COVID-19 phenotypes (base data). To control for potential confounding by ancestry, the target and base data were matched within the same population (EA-EA and AA-AA).COVID-19 PRSs from PRSice-2 were generated using the high-resolution scoring method, which creates PRSs at multiple p-value thresholds in a stepwise manner to find the best-performing set of SNPs in the target data (“Best-PRS”). Best-PRSs were normalized by z-score and their association with AD in the target data was further evaluated in a multivariate logistic regression accounting for age, sex, and *APOE* genotype. A Bonferroni corrected *p*-value ≤ 0.0167 (0.05/3 = 0.0167) from the multivariate logistic regression was used to consider a COVID-19 PRS-AD association statistically significant. In addition, we used linear regression to test if there was any genetic association between best-PRSs of COVID-19 phenotypes and *APOE* genotypes in our EA datasets. Furthermore, a two-sample MR analysis was used to determine the causal effect of COVID-19 on AD using both EA and AA GWAS data.

### 3.2 Data sources

#### 3.2.1 GWAS summary statistics

##### 3.2.1.1 COVID-19 GWASs

COVID-19 GWAS summary statistics data were obtained from Host Genetics Initiative websites in the most updated release 7 (HGI7) (https://www.covid19hg.org/results/r7/, released on April 8, 2022) (18). Three COVID-19 phenotypes included in the study were 1) critical illness (A2); 2) hospitalization (B2); and 3) COVID-19 infection (C2). In the study, we used COVID-19 phenotype GWAS data from EA and AA populations (Table 1). These data were used as base data for PRS calculation in target AD case and control genotype data from their corresponding ancestry groups (see below) and two-sample MR analysis.

##### 3.2.1.2 AD GWASs

We selected summary statistics of two AD GWASs in the EA population to represent the “outcome” in our EA-specific two-sample MR analysis. For a clinically-diagnosed AD phenotype, we selected the AD GWAS by Kunkle *et al* (GCST007511) with 21,982 cases and 41,944 controls (19). We also used a GWAS that combined clinically diagnosed AD cases with “proxy” AD cases who had self-reported at least one parent with AD. This study by Bellenguez *et al*, had a sample size of 39,106 clinically diagnosed AD cases, 46,828 proxy AD cases, and 401,577 controls (20). In the AA population-specific two-sample MR analysis, we used an additional study by Kunkle *et al* (https://www.niagads.org/datasets/ng00100) with a total of 2784 individuals with AD and 5222 controls (21).

#### 3.2.2 Individual-level genotype data (target data) for AD cases and controls

Target data were individual-level genotype data for AD cases and controls in both EA and AA populations. As seen in Table 1, target datasets included the National Institute of Aging/Late-onset Alzheimer’s Disease Study (NIA/LOAD) cohort consents 1–4 (ADc1234) (22), the Multi-Site Collaborative Study for Genotype-Phenotype Associations in Alzheimer’s Disease (GenADA) Study (23), Indianapolis-Ibadan Dementia Project (CIDR) (24), and the Indianapolis African American GWAS (INDY) (25, 26). The first three datasets were downloaded from dbGaP (https://www.ncbi.nlm.nih.gov/gap/), while the last one (INDY) was downloaded from NIAGADS NG00047. As the ADc1234 dataset had both EA and AA participants, we extracted each group as ADc1234EA and ADc1234AA for subsequent analysis. The GenADA dataset was EA only, while CIDR and INDY datasets were AA only. The combined ADc1234EA and GenADA were used as EA samples, whereas the combined ADc1234AA, INDY, and CIDR were for AA samples (Table 1). The AD cases were diagnosed with the criteria proposed in 1984 by the National Institute of Neurological and Communicative Disorders and Stroke and the Alzheimer’s Disease and Related Disorders Association (NINCDS-ADRDA) (27). AD cases were defined as any individual with dementia who was diagnosed with definite, probable, or possible AD at any point in their clinical course (27). Included controls were neurologically evaluated individuals who were age-matched cognitively normal. Unspecified dementia, unconfirmed controls, and controls with other neurological diseases from the original study were removed from this study. The final cases/controls included in each sample were: ADc1234EA, 2,196 cases/2,118 controls; GenADA, 799 cases/778 controls; ADc1234AA, 124 cases/127 controls; INDY, 173 cases/1,002 controls; and CIDR, 85 cases/1,134 controls. Demographic characteristics of all samples were listed in Table 2, along with two major *APOE* SNP genotype information. More detailed descriptions of the data can be found in previous studies (22, 23, 28).

We imputed both EA and AA samples at the Michigan Imputation Server (minimac4) (https://imputationserver.sph.umich.edu) (29) to maximize shared genetic variants across the samples. As a reference, the 1000 Genome Phase 3v5 was utilized (30). Following imputation, the Plink command (--maf 0.01 --hwe 1e-6 --geno 0.01 --mind 0.01) was used to conduct standard quality control (31). The final EA dataset was composed of 5,891 individuals with 8,530,670 SNPs, while the final AA dataset consisted of 2,645 individuals with 12,618,149 SNPs (Table 2).

### 3.3 Polygenic risk score (PRS) calculation and logistic regression analysis

As described in our previous study (13), we constructed PRS calculation using the PRSice-2 software (17). The genetic association between COVID-19 phenotypes and AD in EA and AA populations was evaluated separately with the COVID-19 GWASs as the base data and AD genotype data as the target data. The “best-fit” model implemented in the PRSice-2 was initially used to evaluate the genetic associations between AD and COVID-19 PRSs, with a range of *p*-value thresholds (from 5 × 10^− 8^ to 1.0) and an incremental interval of 0.00005 (--interval 0.00005 --lower 5 × 10^− 8^). Clumping (--clump-kb 250 kb --clump-p 1.0 --clump-r2 0.1) was used to remove SNPs with high linkage disequilibrium (LD). Of note, sample sizes in both AA base and target data were much smaller than those of EA data (Table 1).

To compare the effect sizes in all analyses, we standardized the PRSs from each COVID-19 phenotype into z-scores and conducted the logistic regression analysis using the glm function from the R package stats. The z-scored PRSs did not influence the association *p* values in the regression but provided us with effect sizes as OR for comparison across different phenotypes and ethnic groups. Wilcox test tool in R (v4.2.0) was used to compare the normalized PRSs of three COVID-19 phenotypes between AD patients and controls in the EA and AA populations. The results were visualized with box plots using the ggplot2 function from the R program (v.3.3.6). To account for potential confounding variables in our analysis, multivariate logistic regression with the glm function from the R stats package was conducted by adjusting with sex, age, and *APOE* genotypes (rs429358 and rs7412).

### 3.4 Linear regression analyses

To evaluate potential associations between COVID-19 genetic liability and APOE isoform variants, we used a linear regression between the SNP genotypes (rs429358 and rs7412) and the z-scored best-PRSs of the three different COVID-19 phenotypes in both EA and AA cohorts. The lm function from the R stats package was used for the linear regression to test whether the two SNP genotypes were correlated with the PRSs of COVID-19.

### 3.5 Two-sample Mendelian randomization (MR) analyses

We used two-sample MR to test if there was any causal effect of COVID-19 phenotypes on AD liability with the TwoSampleMR (v.0.6.2) package in R (32). Two-sample MR is used to infer a causal relationship between an exposure trait and outcome trait with a set of SNPs associated with the exposure that are used as instrumental variables (IVs). To be valid IVs, these SNPs must be associated with the exposure, have no independent effects on the outcome, and not be associated with confounding traits of the exposure or outcome (33).

#### 3.5.1 Instrumental variable selection

For the primary MR analysis in the EA population, we used the EA-specific GWAS summary statistics of three COVID-19 phenotypes (infection, hospitalization, and critical illness) as exposures and two EA AD GWASs as outcomes. For the EA population, we selected genome-wide significant SNPs (*p* < 5×10^− 8^) as IVs associated with the 3 COVID phenotypes. For the AA population, SNPs with a *p*-value < 5×10^− 7^ in each COVID phenotype were used as IVs due to fewer SNPs passing the stricter GWAS threshold. To ensure any SNPs used as IVs were independent, we clumped them for linkage disequilibrium over 10,000kB with an r^2^ threshold of 0.01 (clump_kb = 10,000, clump_r^2^ = 0.01) using ancestry-matched LD references from the 1000 Genomes Project for the EA and AA populations (30).

#### 3.5.2 Estimation of causal effects

We used the inverse variance weighted (IVW) (34) regression as the primary method to determine a causal relationship between COVID phenotypes and AD liability. A series of sensitivity analyses were used to test the strength and viability of IVs, as well as the robustness of causal relationships, accounting for potential biases such as pleiotropy and heterogeneity. The MR-Egger regression and intercept test were used to identify horizontal pleiotropy, which is the possibility that IVs influence the outcome trait independently from the exposure trait (35). If the MR-Egger regression causal effect maintained a consistent direction of effect with the IVW regression, and if the intercept test was non-significant (*p* > 0.05), we considered the presence of horizontal pleiotropy to be unlikely. We also used the weighted median regression to rule out the possibility of invalid IVs influencing an observed causal effect as long as its direction of effect was consistent with the IVW estimate (36). Additionally, Cochran’s Q test (37) and the MR-Steiger test (38), were used to assess the effect of heterogeneity on the IVs, and the possibility of reverse causality between exposure and outcome respectively. We used a *p*-value > 0.05 in Cochran’s Q test to indicate no potential heterogeneity, and a r^²^_exposure_ > r^²^_outcome_ in the MR-Steiger test to exclude a possible reverse causal relationship between COVID-19 and AD liability.

### 3.6 Identification of overlapping variants/genes between COVID-19 and AD

To identify shared risk variants and genes between COVID-19 and AD, we compared their respective GWASs (18, 20). We first extracted the shared genome-wide significant SNPs (*p* < 5× 10^− 8^) in the COVID-19 phenotype and AD GWASs from the EA and AA populations. For the EA population, we selected the more recent AD GWAS by Bellenguez *et al* due to its higher sample size (20). We then conducted gene annotation for the SNPs that were genome-wide significant in both GWASs using ANNOVAR (https://annovar.openbioinformatics.org/en/latest/) (39). We selected nonsynonymous SNPs from the shared significant SNPs, and the linkage disequilibrium (LD) structure of the overlapping regions was visualized using the HaploView software 7.0 (40) for the European population from HapMap3 CEU samples (41). Locus plots of SNPs in the chr17q21.31 region were generated at http://locuszoom.sph.umich.edu/.

### 3.7 Phenome-wide association scan (PheWAS)

Phenome-wide association scans (PheWASs) aim to explore the complex genotype-phenotype relationships and pleiotropy between individual SNPs across a large number of different phenotypes (42). We carried out a PheWAS to investigate the role of shared variants and genes between COVID-19 phenotypes and AD in other human phenotypes in the general population. In this study, we scanned for the association of shared significant SNPs and 11 genes in the chr17q21.31 region in the GWASs of 4,756 phenotypes collected in the GWAS ATLAS (https://atlas.ctglab.nl/PheWAS) (43). Among the shared significant SNPs identified in this study, we chose one representative SNP from each of the two major haplotype blocks in the region due to the high LD within each block. Each SNP was genome-wide significant for AD and the COVID phenotypes A2 and B2 in the EA population. The two variants were rs12373123 from *SPPL2C* and rs199515 from *WNT3*. A PheWAS association was considered significant with a Bonferroni corrected *p* < 1.05 ×10^− 5^ (0.05/4,756).

### 3.8 Statistical analyses

COVID-19 PRS associations with LOAD diagnosis with *P <* 0.0167 (0.05/3 with Bonferroni correction) were considered significant for either the EA or AA populations. For other statistical analyses, such as the Wilcoxon Rank Sum test in the boxplot (44) and two-sample MR, *p* < 0.05 was considered significant.

## Results

### 4.1 AD cohort demographics

Demographics of AD datasets are summarized in Table 2. In the AD datasets, the age for each case was defined as the patient’s age at onset (AAO), while the age for each control was the age when the participant was at the first visit for examination (AAE). Notably, the average age for cases was significantly older than that of controls in the three datasets (ADc1234EA, ADc1234AA, and CIDR), while the average age was significantly younger in cases in GenADA dataset. In INDY dataset, the average age was not significantly different between cases and controls. A majority of the participants in the overall datasets were females, representing more than 60% of the samples. For the SNPs in the *APOE* gene, the C allele of rs429358 was significantly higher in cases than in controls in both EA and AA populations; however, the C allele of rs7412 was significantly higher in cases than in controls of EA population but not in AA population (Table 2).

### 4.2 Positive associations between genetic risk of COVID-19 and AD diagnosis in the EA population

In this study, we found that the PRSs of the three COVID-19 phenotypes were all positively associated with AD diagnosis from the “best-fit” model in the EA population (all *p* ≤ 0.0167) (Table 3). COVID-19 infection (C2) PRSs showed the strongest association with AD [OR = 1.142 (1.085–1.203), *p* = 3.90 × 10^−^ 7 at *P*_T_ = 0.042 with 45,462 SNPs], followed by the critical COVID-19 (A2) [OR = 1.099 (1.044–1.157), *p* = 3.03 × 10^− 4^ at *P*_T_ = 0.004 with 7,116 SNPs], then COVID-19 hospitalization (B2) [OR = 1.076 (1.022–1.132), *p* = 5.14 × 10^− 3^ at *P*_T_ = 0.206 with 127,233 SNPs] (Table 3).

Furthermore, we conducted a multivariate logistic regression with demographic covariates (age, sex, and the *APOE* genotypes) to determine any confounding effects on the association between PRSs of COVID-19 and AD. When adjusted for covariates, we found that all positive associations between the three COVID-19 phenotypes and AD remained consistent (Table 3 and Supplementary Table 1). These results indicated that the associations between PRSs of COVID-19 phenotypes and AD diagnosis were independent of age, sex, and the two *APOE* SNP genotypes.

### 4.3 Positive associations between genetic risk of COVID-19 and AD diagnosis in the AA population

Next, we tested whether the associations between COVID-19 PRSs and AD existed in the AA population. We found that critical COVID-19 (A2) PRSs had the strongest positive association with AD, followed by general infection (C2) and hospitalization (B2) PRSs (Table 3). With similar *P*_T_ and comparable numbers of SNPs for the best-PRSs, the association for A2 was OR = 1.721 (1.560–1.902), *p* = 6.13 × 10^− 27^ at *P*_T_ = 0.4198 with 314,341 SNPs, for C2 was OR = 1.310 (1.179–1.456), *p* = 5.21 × 10^− 7^ at *P*_T_ = 0.458 with 378,783 SNPs in C2, and for B2 was OR = 1.260 (1.132–1.402), *p* = 2.47 × 10^− 5^ at *P*_T_ = 0.4 with 346,677 SNPs in B2 (Table 3).

We further conducted multivariate logistic regression analyses adjusted with age, sex, and *APOE* genotypes in the AA population. Again, all the associations remained significant after the adjustment. Notably, the associations in the AA population were much stronger compared to those in the EA population. For example, for COVID-19 A2 ORs = 1.721 (1.560–1.902), *p* = 6.13 × 10^− 27^ in AA population, while OR = 1.099 (1.044–1.157), *p* = 3.03 × 10^− 4^ for EA population in the simple logistic regression (Tables 3). Overall, these results suggested that genetic risk for three different severities of COVID-19 in both EA and AA individuals was highly correlated with AD. This correlation was independent of age, sex, and the *APOE* genotypes.

**Figure 2:**
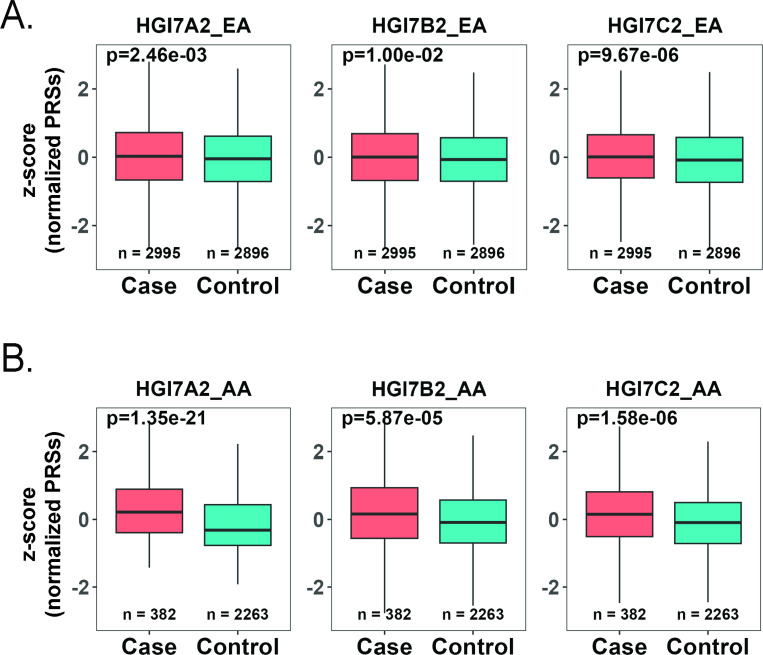
Normalized PRSs of three different COVID-19 phenotypes in AD cases and controls PRSs of COVID-19 were significantly higher in AD cases as compared to AD controls in both the EA (A) and AA populations (B) (*p* < 0.05), suggesting that genetic risk of different COVID-19 severities is likely a risk factor for AD. Wilcoxon Rank Sum test was applied to generate *p* values. X-axis: Diagnosis (AD cases/controls). Y-axis: z-score normalized PRSs of three COVID-19 phenotypes. Of note, correlations between COVID-19 and AD were stronger in AA as compared to EA.

As shown in boxplots in both EA (Fig. 2A) and AA (Fig. 2B) populations, AD patients exhibited higher PRSs for COVID-19, as compared to cognitively normal controls in all three COVID-19 phenotypes (A2, B2, and C2). Overall, we found that the associations were stronger in AA populations than in EA populations. For both populations adjusted with age, sex, and *APOE* genotypes (Supplementary Tables 1 and 2), age and *APOE* rs429358 minor allele C were significant risk factors for AD. For the *APOE* rs7412 minor allele T, we detected a protective effect for AD in the EA population, but not in the AA population. Sex tended to have no significant contribution to the genetic correlations in either EA or AA populations in this study.

### 4.4 Association of *APOE* with COVID-19 PRSs

The human *APOE* gene has three alleles (ε2, ε3, and ε4) based on the genotypes of SNPs rs429358 and rs7412, among which ε4 is the strong genetic risk factor for AD. In this study, we did not find any correlation between the two *APOE* SNPs and the PRSs of the three different COVID-19 phenotypes in either EA or AA populations (Supplementary Table 3).

### 4.5 No causal effects of COVID-19 phenotypes on AD

**Figure 3:**
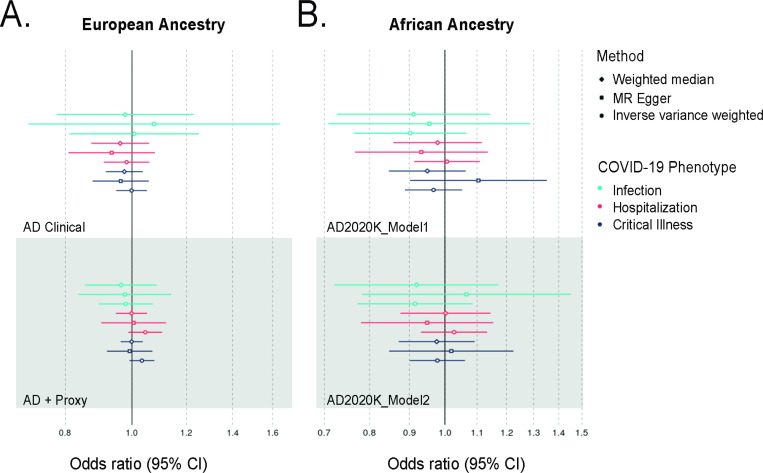
Two-sample Mendelian randomization of COVID-19 phenotype exposures and AD Outcomes Filled circles on forest plot indicate a significant (*p* < 0.05) causal effect. A. European-ancestry MR results. AD Clinical: Clinically diagnosed AD GWAS by IGAP and Kunkle *et al.* (2019) used as an outcome. AD + Proxy: Clinically diagnosed and Proxy AD GWAS by Bellenguez *et al.* (2022) used as an outcome. B. African-ancestry MR results. AD2020K_Model1: AD GWAS by Kunkle *et al.* (2020) with associations adjusted for age, sex, and genetic principal components used as an outcome. AD2020K_Model2: AD GWAS by Kunkle *et al.*(2020) with associations adjusted for model 1 covariates and APOE genotype used as an outcome.

We used two-sample MR to estimate the causal effect of the 3 different phenotypes of COVID-19 (critical illness A2, hospitalization B2, and infection C2) on two AD GWASs in the EA population and one AD GWAS in the AA population. Our goal was to see if the positive associations seen between PRSs of the COVID-19 phenotypes with AD were translatable to causal relationships under the stricter assumptions and requirements of MR. Using IVW regression, we found no significant causal relationships between any COVID-19 phenotype and AD liability in the EA population (Fig. 3A). This null relationship held regardless of whether the AD GWAS used as the outcome contained “proxy” AD cases, a measure of self-reported family history of AD utilized to improve the sample sizes of AD GWASs (Supplementary Table 4) (45). In both AD phenotypic outcomes, the COVID-19 critical illness and hospitalization exposures had significantly heterogeneous IVs as measured by Cochran’s Q test (Supplementary Table 4). When tested in the AA population, there was again no causal effect of any COVID-19 phenotype on AD liability (Fig. 3B). These results suggest a consistently null effect of COVID-19 susceptibility, hospitalization, or critical illness on AD liability in both EA and AA populations.

### 4.6 Overlapping significant SNPs/genes and PheWAS suggest pleiotropy between COVID-19 phenotypes and AD

Comparative analysis of GWAS summary statistics between COVID-19 phenotypes and the combined clinical/proxy AD phenotype revealed substantial genomic overlap despite the absence of direct causal relationships. We identified 957 genome-wide significant SNPs shared between AD and COVID-19 critical illness (A2), and 1,412 between AD and COVID-19 hospitalization (B2) in the EA population (Supplementary Table 5). No shared variants meeting genome-wide significance criteria were identified between AD and COVID-19 infection susceptibility (C2), suggesting phenotype-specific genetic architecture. In addition, there were no genome-wide significant SNPs shared between AD and any phenotypes of COVID-19 in the AA population, but this is likely due to the lower sample size of the AA-AD GWAS (Supplementary Table 6).

**Figure 4:**
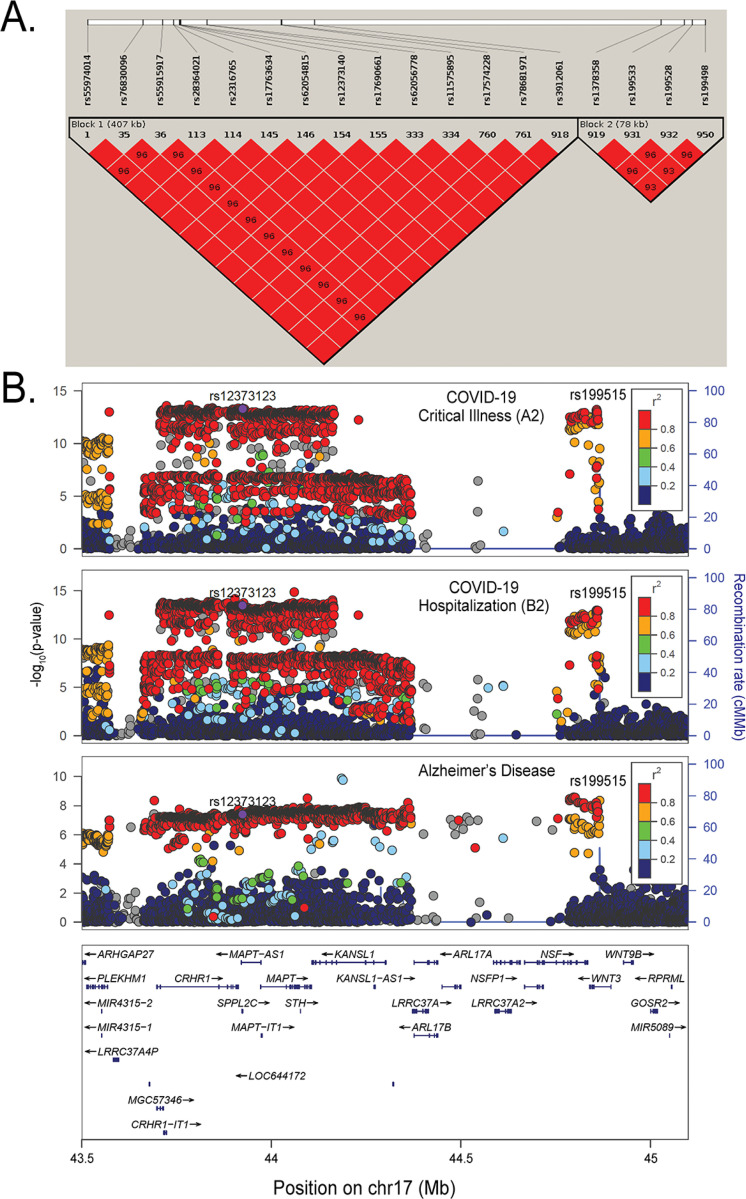
LD structure and SNP significance in the shared chr17q21.31 region **A.** LD blocks across SNPs at chr17q21.31 in the EA population. **B.** Locus plots of SNPs centered around rs12373123 and rs199515 including their LD r^2^, chromosomal position, and p-value cross the critical illness (A2), hospitalization (B2) COVID-19 GWASs, and AD GWAS by Bellenguez *et al*.

We used ANNOVAR to map the shared SNPs to 11 protein-coding genes including *CRHR1*, *SPPL2C*, *MAPT*, *STH*, *KANSL1*, *ARL17B*, *LRRC37A*, *LRRC37A2*, *ARL17A*, *NSF*, and *WNT3* in the chr17q21.31 region. Among these overlapping genes, nonsynonymous SNPs in the exons across *SPPL2C*, *CRHR1*, *MAPT*, *STH*, and *KANSL1* genes were identified, among which, 13 were from COVID-19 critical illness and 15 were from COVID-19 hospitalization (Supplementary Table 7). Examining the LD in this shared region showed that there were at least two independent blocks of high LD among these significant SNPs, with the first block covering 9 genes (*CRHR1*, *SPPL2C*, *MAPT*, *STH*, *KANSL1*, *ARL17B*, *LRRC37A*, *LRRC37A2*, and *ARL17A*), and the second block covering two genes (*NSF* and *WNT3*) (Fig. 4A). Locus plots of SNPs in the chr17q21.31 region across the COVID-19 phenotypes and AD were visualized in Fig. 4B.

We next performed a PheWas, which showed that SNPs in the chr17q21.31 region were associated with multiple phenotypes besides COVID-19 and AD. For example, rs12373123 from the *SPPL2C* and rs199515 from the *WNT3* genes were significantly associated with multiple phenotypes. These include psychiatric and immunological domains, as well as lung function phenotypes such as forced vital capacity (FVC) and forced expiratory volume in 1-second (FEV1) (Supplementary Tables 8 and 9) (46). These results show that the genomic region shared between COVID-19 hospitalization, critical illness, and AD + proxy AD phenotypes is highly pleiotropic across different traits, which likely influenced the observed heterogeneity in SNPs selected as IVs in two-sample MR.

## Discussion

This comprehensive genetic epidemiology investigation demonstrated significant polygenic associations between COVID-19 severity phenotypes and Alzheimer’s disease across ancestral populations. Through integration of large-scale GWAS datasets with individual-level genotype data, we identified robust associations between COVID-19 polygenic risk scores and AD diagnosis in both European and African ancestry cohorts. These associations remained significant after adjusting for age, sex, and the *APOE*-e4 genotype of individuals, and tended to be stronger in the AA population than the EA population. However, when each COVID-19 phenotype was used as an exposure with AD as an outcome in a two-sample MR analysis, we were unable to identify a causal relationship between the traits. There was no significant causal effect of the COVID-19 phenotypes when the AD outcome included or excluded “proxy” AD cases in the EA analysis, and no significant effect was seen in the AA population. Further analysis of overlapping SNPs between COVID-19 hospitalization and critical illness with AD in the EA population revealed a shared genomic region (chr17q21.31) with genome-wide significant SNPs in both GWASs. A PheWAS of several SNPs in this region revealed associations with phenotypes related to lung function, immune function, and psychiatric traits. This is indicative of a polygenic overlap between the traits that is driven by a highly pleiotropic genomic region involved in multiple human phenotypes.

A widespread issue in applications of GWAS to risk-score prediction and determining causal relationships is that most top GWAS hits explain little heritability in the traits they cover- the “missing heritability” problem (47). Pritchard *et al* have proposed that for polygenic traits there is a distinction between the “core” genes directly contributing to the development of the trait and the “peripheral” genes that contribute most to the heritability of that trait (the “Omnigenic” model) (48). They propose that gene regulatory networks are sufficiently interconnected so that many small trans-QTL effects can mediate the effects that “core” and “peripheral” genes have on the traits with which they are associated in a GWAS (49). Our contrasting findings of an association between PRSs of COVID-19 phenotypes and AD, but a lack of a causal effect between them, suggests that they may share a broader polygenic background, but lack a direct relationship between highly-associated genes that are involved in the most proximal disease pathways.

In contrast to our two-sample MR results, several prior MR studies have found that genetically predicted hospitalized and critically ill COVID-19 liability carries an increased risk of AD (50, 51). However, it should be noted that these studies did not match the EA population of the AD GWASs used as “outcomes” with the COVID-19 GWASs used as “exposures”, instead opting to use the COVID-19 GWASs from all ancestry groups combined. By doing MR analyses in an ancestry-specific manner, our approach avoided potential genetic confounding from the mismatch of ancestries. In addition, by using multiple GWASs of AD, we were able to show that the inferred causal relationship between COVID-19 infection and AD was robust to the phenotypic differences between AD + proxy AD GWAS and clinically diagnosed AD (52).

Our study has several strengths. First, using the most recent COVID-19 phenotype and AD GWASs allowed us greater power to detect associations between COVID-19 PRSs and AD that were independent of age, sex, or *APOE* genotypes. This increase in statistical power also allowed us to discover that there were likely no causal effects of the COVID-19 phenotypes on AD through the application of two-sample MR. This helps us understand how shared genetic factors impact COVID-19 phenotypes and AD in the post-pandemic era. Second, our analysis included GWASs and genotype data from diverse groups, which aimed to understand how the genetic risk factors intersect with disease outcomes in different genetic backgrounds. Including diverse populations in studies helps to reduce existing inequalities and health disparities among different groups. It is worth noting that the COVID-19 PRS associations with AD were much stronger in the AA cohort compared to the EA cohort. This result was consistent with other observational studies that the COVID-19 pandemic has differentially impacted AA populations (53). Our study has several limitations. Notably, the sample size for AA population cohorts was much smaller than that for the EA population, which may explain why we did not detect any significant SNPs shared between COVID-19 and AD in this group. We also lacked information on comorbidities that could have influenced either COVID-19 or AD, such as diabetes, cardiovascular diseases, and obesity. Future studies with more diverse ethnic groups and phenotypic information are essential to understand how they may impact the complex genetic architecture between the two traits. This would also help to understand if prior associations between COVID-19-associated neuronal damage and neurological diseases have more to do with nongenetic factors and the environment.

Overall, our study shows that COVID-19 phenotypes and AD have a genetic relationship that is more driven by polygenic overlap than a direct causal relationship mediated through shared disease pathways. Future investigations with large cohorts of AD and COVID-19 patients from different ethnic backgrounds are needed to better understand the pathological relationship between the two diseases and potential targets for better prevention and treatment.

## Supplementary Material

Supplement 1

## Abbreviations

COVID-19: Coronavirus disease 2019
AD: Alzheimer’s disease
PRS: Polygenic Risk Score
GWAS: Genome-wide association study
EA: European Ancestry
AA: African Ancestry
HGI7: Host Genetics Initiative release 7
A2: Critical illness of COVID-19
B2: Hospitalization COVID-19
C2: COVID-19 infection
OR: Odds Ratio
CI: Confidence Interval
CNS: Central Nervous System
ARDS: acute respiratory distress syndrome
SNP: Single Nucleotide Polymorphism
IRB: Institutional Review Board
NIA/LOAD: National Institute of Aging/Late-onset Alzheimer’s Disease Study
ADc1234: National Institute of Aging/Late-onset Alzheimer’s Disease Study cohort consents 1–4
GenADA: Multi-Site Collaborative Study for Genotype-Phenotype Associations in Alzheimer’s Disease
CIDR: Indianapolis-Ibadan, Nigeria Comparative Epidemiological Study of Dementia
INDY: Indianapolis African American GWAS
LD: Linkage Disequilibrium
MR: Mendelian randomization
AAO: Age at Onset
AAE: Age at Examination
ACE2: Angiotensin-converting enzyme 2
NINCDS-ADRDA: National Institute of Neurological and Communicative Disorders and Stroke and the Alzheimer’s Disease and Related Disorders Association
UNLV: University of Nevada Las Vegas

## References

[1] Arvanitakis Z, Shah RC, Bennett DA (2019) Diagnosis and Management of Dementia. Rev JAMA 322(16):1589 10.1001/jama.2019.4782 PMC7462122 31638686

[2] (2022) Alzheimer’s disease facts and figures. Alzheimers Dement. 2022;18(4):700–89 35289055 10.1002/alz.12638

[3] Nichols E, Steinmetz JD, Vollset SE, Fukutaki K, Chalek J, Abd-Allah F (2022) Estimation of the global prevalence of dementia in 2019 and forecasted prevalence in 2050: an analysis for the Global Burden of Disease Study 2019. Lancet Public Health 7(2):e105–e125 34998485 10.1016/S2468-2667(21)00249-8 PMC8810394

[4] Cummings JL, Morstorf T, Zhong K (2014) Alzheimer’s disease drug-development pipeline: few candidates, frequent failures. Alzheimers Res Ther 6(4):37 25024750 10.1186/alzrt269 PMC4095696

[5] Itzhaki RF, Golde TE, Heneka MT, Readhead B (2020) Do infections have a role in the pathogenesis of Alzheimer disease? Nat Rev Neurol 16(4):193–197 32152461 10.1038/s41582-020-0323-9

[6] Ciaccio M, Lo Sasso B, Scazzone C, Gambino CM, Ciaccio AM, Bivona G (2021) COVID-19 and Alzheimer’s Disease. Brain Sci 11(3):305 33673697 10.3390/brainsci11030305 PMC7997244

[7] Bougakov D, Podell K, Goldberg E (2021) Multiple Neuroinvasive Pathways in COVID-19. Mol Neurobiol 58(2):564–575 32990925 10.1007/s12035-020-02152-5 PMC7523266

[8] Beckman D, Bonillas A, Diniz GB, Ott S, Roh JW, Elizaldi SR (2022) SARS-CoV-2 infects neurons and induces neuroinflammation in a non-human primate model of COVID-19. Cell Rep 41(5):111573 36288725 10.1016/j.celrep.2022.111573 PMC9554328

[9] VanElzakker MB, Bues HF, Brusaferri L, Kim M, Saadi D, Ratai EM (2024) Neuroinflammation in post-acute sequelae of COVID-19 (PASC) as assessed by [11C]PBR28 PET correlates with vascular disease measures. Brain Behav Immun 119:713–723 38642615 10.1016/j.bbi.2024.04.015 PMC11225883

[10] Wang L, Davis PB, Volkow ND, Berger NA, Kaelber DC, Xu R (2022) Association of COVID-19 with New-Onset Alzheimer’s Disease. J Alzheimers Dis JAD 89(2):411–414 35912749 10.3233/JAD-220717 PMC10361652

[11] Carriedo A, Cecchini JA, Fernandez-Rio J, Méndez-Giménez A (2020) COVID-19, Psychological Well-being and Physical Activity Levels in Older Adults During the Nationwide Lockdown in Spain. Am J Geriatr Psychiatry Off J Am Assoc Geriatr Psychiatry 28(11):1146–1155 10.1016/j.jagp.2020.08.007 PMC7443087 32919872

[12] Choi SW, Mak TSH, O’Reilly PF (2020) Tutorial: a guide to performing polygenic risk score analyses. Nat Protoc 15(9):2759–2772 32709988 10.1038/s41596-020-0353-1 PMC7612115

[13] Cammann D, Lu Y, Cummings MJ, Zhang ML, Cue JM, Do J (2023) Genetic correlations between Alzheimer’s disease and gut microbiome genera. Sci Rep 13(1):5258 37002253 10.1038/s41598-023-31730-5 PMC10066300

[14] Cammann DB, Lu Y, Rotter JI, Wood AC, Chen J (2024) Polygenic scores and Mendelian randomization identify plasma proteins causally implicated in Alzheimer’s disease. Front Neurosci [Internet]. Jul 23 [cited 2024 Aug 19];18. Available from: https://www.frontiersin.org/journals/neuroscience/articles/10.3389/fnins.2024.1404377/full 10.3389/fnins.2024.1404377 PMC11300309 39108314

[15] van der Linden RJ, De Witte W, Poelmans G (2021) Shared Genetic Etiology between Alzheimer’s Disease and Blood Levels of Specific Cytokines and Growth Factors. Genes 12(6):865 34198788 10.3390/genes12060865 PMC8226721

[16] Handy A, Lord J, Green R, Xu J, Aarsland D, Velayudhan L (2021) Assessing Genetic Overlap and Causality Between Blood Plasma Proteins and Alzheimer’s Disease. J Alzheimers Dis 83(4):1825–1839 34459398 10.3233/JAD-210462 PMC8609677

[17] Choi SW, O’Reilly PF (2019) PRSice-2: Polygenic Risk Score software for biobank-scale data. GigaScience 8(7):giz082 31307061 10.1093/gigascience/giz082 PMC6629542

[18] Kanai M, Andrews SJ, Cordioli M, Stevens C, Neale BM, Daly M (2023) A second update on mapping the human genetic architecture of COVID-19. Nature 621(7977):E7–26 37674002 10.1038/s41586-023-06355-3 PMC10482689

[19] Kunkle BW, Grenier-Boley B, Sims R, Bis JC, Damotte V, Naj AC (2019) Genetic meta-analysis of diagnosed Alzheimer’s disease identifies new risk loci and implicates Aβ, tau, immunity and lipid processing. Nat Genet 51(3):414–430 30820047 10.1038/s41588-019-0358-2 PMC6463297

[20] Bellenguez C, Küçükali F, Jansen IE, Kleineidam L, Moreno-Grau S, Amin N (2022) New insights into the genetic etiology of Alzheimer’s disease and related dementias. Nat Genet 54(4):412–436 35379992 10.1038/s41588-022-01024-z PMC9005347

[21] Kunkle BW, Schmidt M, Klein HU, Naj AC, Hamilton-Nelson KL, Larson EB (2021) Novel Alzheimer Disease Risk Loci and Pathways in African American Individuals Using the African Genome Resources Panel. JAMA Neurol 78(1):1–13 33074286 10.1001/jamaneurol.2020.3536 PMC7573798

[22] Lee JH, Cheng R, Graff-Radford N, Foroud T, Mayeux R, National Institute on Aging Late-Onset Alzheimer’s Disease Family Study Group (2008) Analyses of the National Institute on Aging Late-Onset Alzheimer’s Disease Family Study: implication of additional loci. Arch Neurol 65(11):1518–1526 19001172 10.1001/archneur.65.11.1518 PMC2694670

[23] Li H, Wetten S, Li L, St. Jean PL, Upmanyu R, Surh L (2008) Candidate Single-Nucleotide Polymorphisms From a Genomewide Association Study of Alzheimer Disease. Arch Neurol 65(1):45–53 17998437 10.1001/archneurol.2007.3

[24] Ogunniyi A, Baiyewu O, Gureje O, Hall KS, Unverzagt F, Siu SH (2000) Epidemiology of dementia in Nigeria: results from the Indianapolis-Ibadan study. Eur J Neurol 7(5):485–490 11054131 10.1046/j.1468-1331.2000.00124.x

[25] Reitz C, Jun G, Naj A, Rajbhandary R, Vardarajan BN, Wang LS (2013) Variants in the ATP-Binding Cassette Transporter (ABCA7), Apolipoprotein E ϵ4, and the Risk of Late-Onset Alzheimer Disease in African Americans. JAMA 309(14):1483 23571587 10.1001/jama.2013.2973 PMC3667653

[26] Murrell JR, Price B, Lane KA, Baiyewu O, Gureje O, Ogunniyi A (2006) Association of Apolipoprotein E Genotype and Alzheimer Disease in African Americans. Arch Neurol 63(3):431 16533971 10.1001/archneur.63.3.431 PMC3203415

[27] McKhann G, Drachman D, Folstein M, Katzman R, Price D, Stadlan EM (1984) Clinical diagnosis of Alzheimer’s disease. Neurology 34(7):939 6610841 10.1212/wnl.34.7.939

[28] European Alzheimer’s Disease Initiative (EADI), Genetic and Environmental Risk in Alzheimer’s Disease (GERAD), Alzheimer’s Disease Genetic Consortium (ADGC), Cohorts for Heart and Aging Research in Genomic Epidemiology (CHARGE), Lambert JC, Ibrahim-Verbaas CA (2013) Meta-analysis of 74,046 individuals identifies 11 new susceptibility loci for Alzheimer’s disease. Nat Genet. ;45(12):1452–8 24162737 10.1038/ng.2802 PMC3896259

[29] Das S, Forer L, Schönherr S, Sidore C, Locke AE, Kwong A (2016) Next-generation genotype imputation service and methods. Nat Genet 48(10):1284–1287 27571263 10.1038/ng.3656 PMC5157836

[30] Auton A, Abecasis GR, Altshuler DM, Durbin RM, Abecasis GR, Bentley DR (2015) A global reference for human genetic variation. Nature 526(7571):68–74 26432245 10.1038/nature15393 PMC4750478

[31] Chang CC, Chow CC, Tellier LC, Vattikuti S, Purcell SM, Lee JJ (2015) Second-generation PLINK: rising to the challenge of larger and richer datasets. GigaScience 4(1):s13742-015-0047-8 10.1186/s13742-015-0047-8 PMC4342193 25722852

[32] Hemani G, Zheng J, Elsworth B, Wade KH, Haberland V, Baird D (2018) The MR-Base platform supports systematic causal inference across the human phenome. Loos R, editor. eLife. ;7:e34408 10.7554/eLife.34408 PMC5976434 29846171

[33] Davies NM, Holmes MV, Smith GD (2018) Reading Mendelian randomisation studies: a guide, glossary, and checklist for clinicians. BMJ 362:k601 30002074 10.1136/bmj.k601 PMC6041728

[34] Burgess S, Davey Smith G, Davies NM, Dudbridge F, Gill D, Glymour MM (2019) Guidelines for performing Mendelian randomization investigations: update for summer 2023. Wellcome Open Res 4:186 32760811 10.12688/wellcomeopenres.15555.1 PMC7384151

[35] Bowden J, Davey Smith G, Burgess S (2015) Mendelian randomization with invalid instruments: effect estimation and bias detection through Egger regression. Int J Epidemiol 44(2):512–525 26050253 10.1093/ije/dyv080 PMC4469799

[36] Bowden J, Davey Smith G, Haycock PC, Burgess S (2016) Consistent Estimation in Mendelian Randomization with Some Invalid Instruments Using a Weighted Median Estimator. Genet Epidemiol 40(4):304–314 27061298 10.1002/gepi.21965 PMC4849733

[37] Bowden J, Del Greco MF, Minelli C, Zhao Q, Lawlor DA, Sheehan NA (2019) Improving the accuracy of two-sample summary-data Mendelian randomization: moving beyond the NOME assumption. Int J Epidemiol 48(3):728–742 30561657 10.1093/ije/dyy258 PMC6659376

[38] Hemani G, Tilling K, Davey Smith G (2017) Orienting the causal relationship between imprecisely measured traits using GWAS summary data. PLoS Genet 13(11):e1007081 29149188 10.1371/journal.pgen.1007081 PMC5711033

[39] Wang K, Li M, Hakonarson H (2010) ANNOVAR: functional annotation of genetic variants from high-throughput sequencing data. Nucleic Acids Res 38(16):e164–e164 20601685 10.1093/nar/gkq603 PMC2938201

[40] Barrett JC, Fry B, Maller J, Daly MJ (2005) Haploview: analysis and visualization of LD and haplotype maps. Bioinformatics 21(2):263–265 15297300 10.1093/bioinformatics/bth457

[41] International HapMap 3 Consortium, Altshuler DM, Gibbs RA, Peltonen L, Altshuler DM, Gibbs RA (2010) Integrating common and rare genetic variation in diverse human populations. Nature 467(7311):52–58 20811451 10.1038/nature09298 PMC3173859

[42] Pendergrass SA, Brown-Gentry K, Dudek SM, Torstenson ES, Ambite JL, Avery CL (2011) The Use of Phenome-Wide Association Studies (PheWAS) for Exploration of Novel Genotype-Phenotype Relationships and Pleiotropy Discovery. Genet Epidemiol 35(5):410–422 21594894 10.1002/gepi.20589 PMC3116446

[43] Watanabe K, Stringer S, Frei O, Umićević Mirkov M, de Leeuw C, Polderman TJC (2019) A global overview of pleiotropy and genetic architecture in complex traits. Nat Genet 51(9):1339–1348 31427789 10.1038/s41588-019-0481-0

[44] Wilcoxon F (1945) Individual Comparisons by Ranking Methods. Int Biom Soc 1:80–83

[45] Liu JZ, Erlich Y, Pickrell JK (2017) Case–control association mapping by proxy using family history of disease. Nat Genet 49(3):325–331 28092683 10.1038/ng.3766

[46] Sinkala M, Elsheikh SSM, Mbiyavanga M, Cullinan J, Mulder NJ (2023) A genome-wide association study identifies distinct variants associated with pulmonary function among European and African ancestries from the UK Biobank. Commun Biol 6:49 36641522 10.1038/s42003-023-04443-8 PMC9840173

[47] Matthews LJ, Turkheimer E (2022) Three Legs of the Missing Heritability Problem. Stud Hist Philos Sci 93:183 35533541 10.1016/j.shpsa.2022.04.004 PMC9172633

[48] Boyle EA, Li YI, Pritchard JK (2017) An Expanded View of Complex Traits: From Polygenic to Omnigenic. Cell 169(7):1177–1186 28622505 10.1016/j.cell.2017.05.038 PMC5536862

[49] Liu X, Li YI, Pritchard JK (2019) Trans Effects on Gene Expression Can Drive Omnigenic Inheritance. Cell 177(4):1022–1034e6 31051098 10.1016/j.cell.2019.04.014 PMC6553491

[50] Baranova A, Cao H, Zhang F (2022) Causal effect of COVID-19 on Alzheimer’s disease: A Mendelian randomization study. J Med Virol. ;jmv.28107 10.1002/jmv.28107 PMC9539282 36039844

[51] Tirozzi A, Santonastaso F, de Gaetano G, Iacoviello L, Gialluisi A (2022) Does COVID-19 increase the risk of neuropsychiatric sequelae? Evidence from a mendelian randomization approach. World J Psychiatry 12(3):536–540 35433322 10.5498/wjp.v12.i3.536 PMC8968503

[52] Wu Y, Sun Z, Zheng Q, Miao J, Dorn S, Mukherjee S (2024) Pervasive biases in proxy genome-wide association studies based on parental history of Alzheimer’s disease. Nat Genet. ;1–8 39496879 10.1038/s41588-024-01963-9 PMC11929606

[53] Tai DBG, Shah A, Doubeni CA, Sia IG, Wieland ML (2020) The Disproportionate Impact of COVID-19 on Racial and Ethnic Minorities in the United States. Clin Infect Dis Off Publ Infect Dis Soc Am. ;ciaa815 10.1093/cid/ciaa815 PMC7337626 32562416

