# Supplementary material for: Shared Genetic Architecture Between COVID-19 Severity and Alzheimer’s Disease Across European and African Ancestries
Source: Res Sq. 2024 Dec 24:rs.3.rs-5619229. Preprint. [Version 1] doi: 10.21203/rs.3.rs-5619229/v1 (PMC11703345; doi:10.21203/rs.3.rs-5619229/v1)
Supplement: Supplement 1 [file NIHPPRS5619229V1-supplement-1.pdf]

Supplementary Files

This is a list of supplementary files associated with this preprint. Click to download.

- SupplementaryTables.xlsx
- Tables13TransPsychCopy.xlsx
